# A Computational Model of Aging and Calcification in the Aortic Heart Valve

**DOI:** 10.1371/journal.pone.0005960

**Published:** 2009-06-18

**Authors:** Eli J. Weinberg, Frederick J. Schoen, Mohammad R. K. Mofrad

**Affiliations:** 1 Molecular Cell Biomechanics Laboratory, Department of Bioengineering, University of California, Berkeley, California, United States of America; 2 Departments of Pathology, Brigham and Women's Hospital and Harvard Medical School, Boston, Massachusetts, United States of America; 3 Harvard-MIT Division of Health Sciences and Technology, Cambridge, Massachusetts, United States of America; Istituto Dermopatico dell'Immacolata, Italy

## Abstract

The aortic heart valve undergoes geometric and mechanical changes over time. The cusps of a normal, healthy valve thicken and become less extensible over time. In the disease calcific aortic stenosis (CAS), calcified nodules progressively stiffen the cusps. The local mechanical changes in the cusps, due to either normal aging or pathological processes, affect overall function of the valve. In this paper, we propose a computational model for the aging aortic valve that connects local changes to overall valve function. We extend a previous model for the healthy valve to describe aging. To model normal/uncomplicated aging, leaflet thickness and extensibility are varied versus age according to experimental data. To model calcification, initial sites are defined and a simple growth law is assumed. The nodules then grow over time, so that the area of calcification increases from one model to the next model representing greater age. Overall valve function is recorded for each individual model to yield a single simulation of valve function over time. This simulation is the first theoretical tool to describe the temporal behavior of aortic valve calcification. The ability to better understand and predict disease progression will aid in design and timing of patient treatments for CAS.

## Introduction

Aging of the aortic valve (AV) is characterized by cuspal thickening [1] and loss of extensibility [2], which can lead to progressive changes in valve function with age, but these are not usually themselves of clinical significance. The most common disease of the AV is calcific aortic stenosis (CAS), found in 2% of individuals over 65 years and in 4% of those over 85 [3]. Early lesions with some features of atherosclerosis are found in almost all adults [4].These lesions may progress into calcified nodules, which can grow over time, stiffening the valve leaflets and eventually critically interfering with valve opening and potentially closing [5]. Currently, the most common treatment for CAS is valve replacement with a mechanical or bioprosthetic valve [6]. CAS is the leading single etiology of valve disease necessitating replacement, accounting for a major fraction of the approximately 300,000 valve replacement surgeries worldwide each year [7].

Overall valve function depends on the mechanical properties of the cuspal tissue: stiffer, thicker tissue causes the valve to be less efficient. A model that describes the connection between tissue properties and valve function will be clinically useful in two ways. First, such a model can be used in conjunction with existing imaging techniques to improve diagnostic criteria and to aid in making decisions regarding timing of existing surgical therapies. Second, such a model could be used to quantify the effects of calcification on valve function and to aid in the design of treatments aimed at preventing CAS onset and delaying valve failure once CAS is present.

Methods presently used in deciding when to intervene involve examination both of the valve function and the state of the tissue. Valve function is evaluated by using chest imaging to measure various properties of blood flow [8]–[11] and various geometric parameters of the valve [12]–[14]. Calcification is examined by cardiac catheterization [12], [15] or, more recently, chest imaging [16]. A model that incorporates both valve function and tissue health could aid in predicting the course of disease and in deciding when to intervene.

In addition to aiding decision-making regarding existing procedures, a model of calcific disease could be useful in examining and designing emerging methods. Since the loss of valve function is due to tissue dysfunction, treatments to prevent or slow disease progression must target the tissue. Current options for preventing the onset of CAS or valve failure are limited; pharmaceutical approaches such as statins or other drugs may ultimately be useful but have not shown consistent benefit in prior studies [17]. A better understanding of the tissue-based nature of CAS progression will enhance our ability to develop new pharmaceutical and surgical treatments.

In this paper, we create a model for valve aging which describes the impact of changes to tissue properties on valve function. We have previously described a multiscale simulation of the healthy aortic valve [18], where we modeled the valve at one point in the patient's lifetime. In the present paper, we extend the simulation to model ages from 20 to 80. This collection of simulations describes aging in the aortic valve, including calcification, over a patient's adult life.

## Results

**Figure 1:**
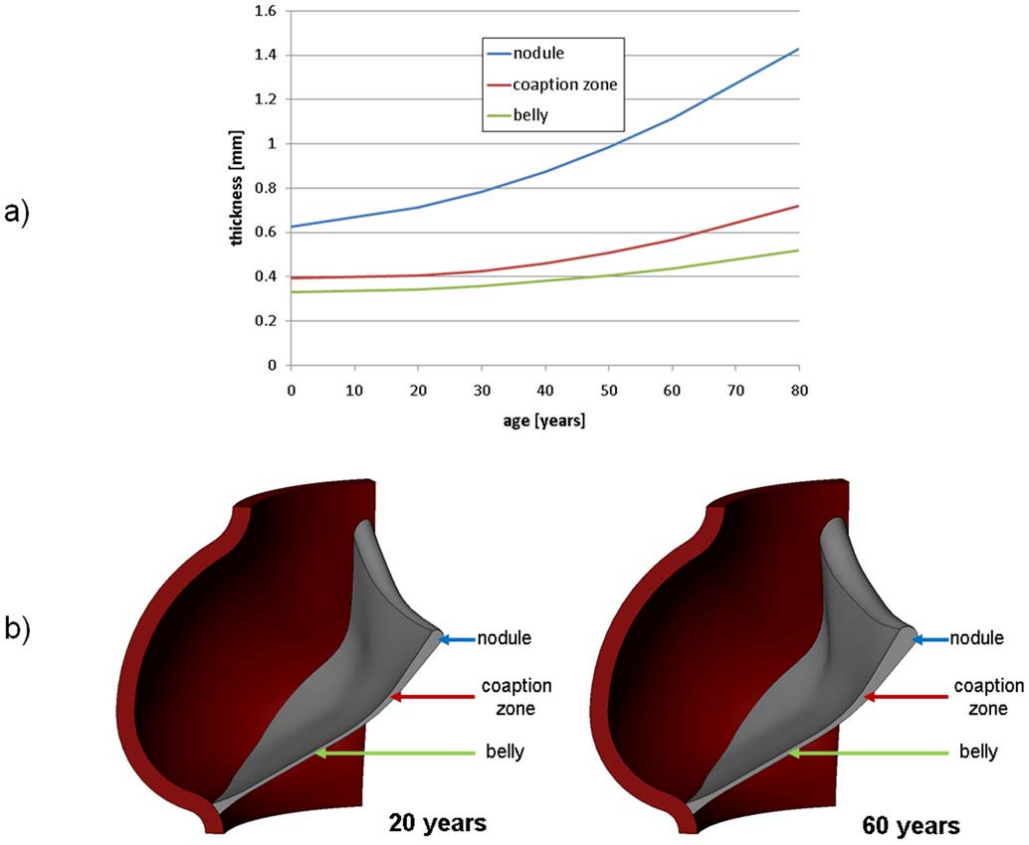
Changes in valve geometry with age. a) Measured thickness variation (Sahasakul 1988) and b) CAD geometry at ages 20 and 60 years.

**Figure 2:**
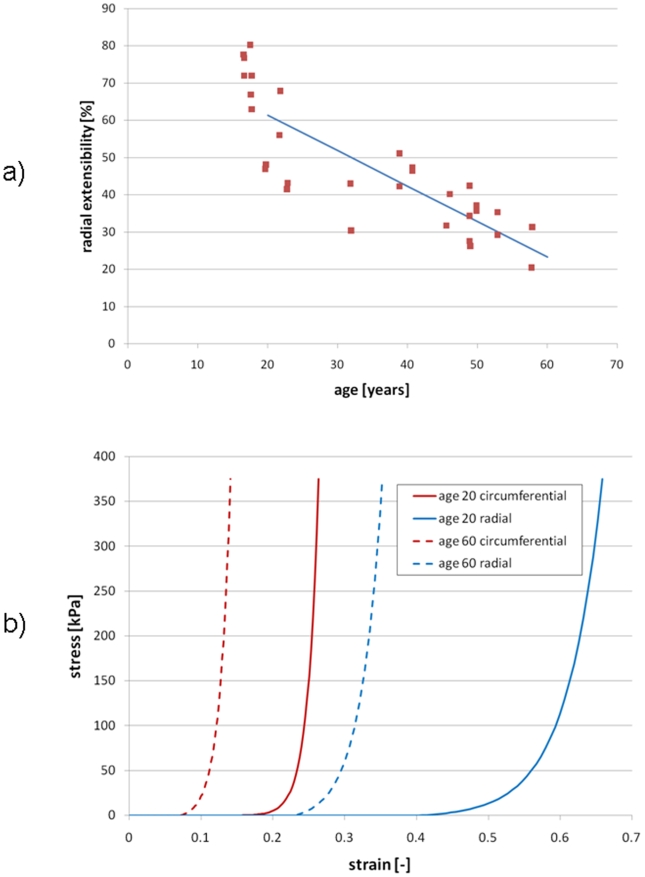
Changes in leaflet extensibility with age (Christie 1995). a) Measured radial extensibility versus age and b) Circumferential and radial extensibilities used in model at ages 20 and 60.

**Figure 3:**
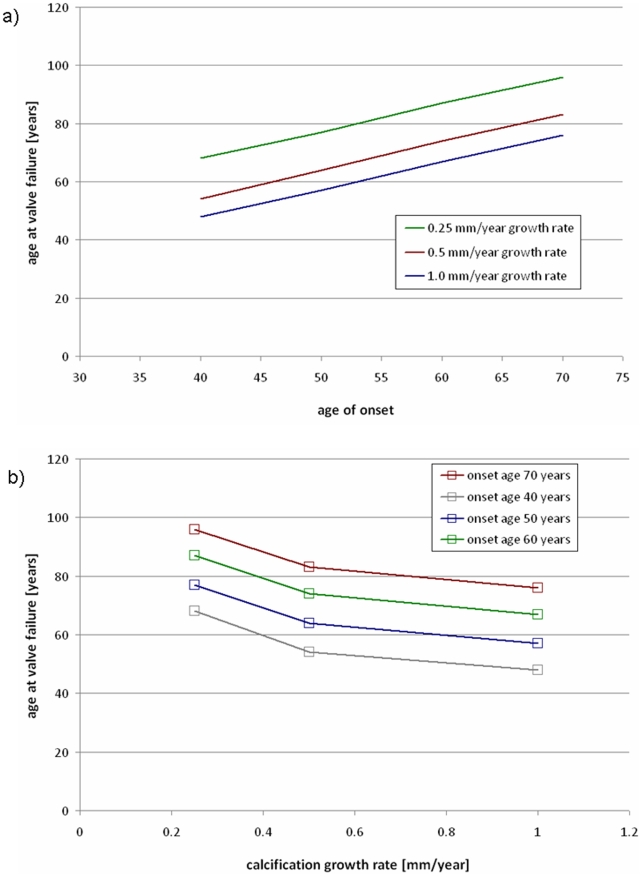
Simulated growth of calcified nodules.

**Figure 4:**
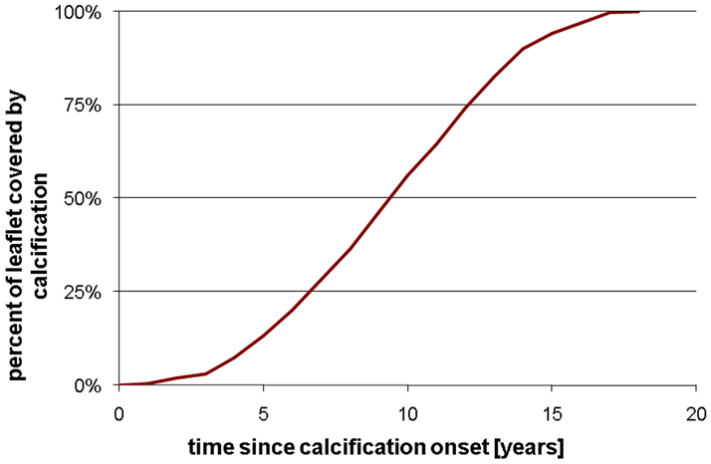
Percent of leaflet covered by calcification versus time.

A number of computational models were created to simulate aging in the human aortic valve, with and without calcification (see Figures 1, 2, 3, 4 and **Methods** for illustration and detailed description of these simulations). All simulations ran to convergence with no computational instabilities. Computation time was approximately 3 hours per cardiac cycle on a workstation with four Xeon 5160 3.00 GHz processors.

**Figure 5:**
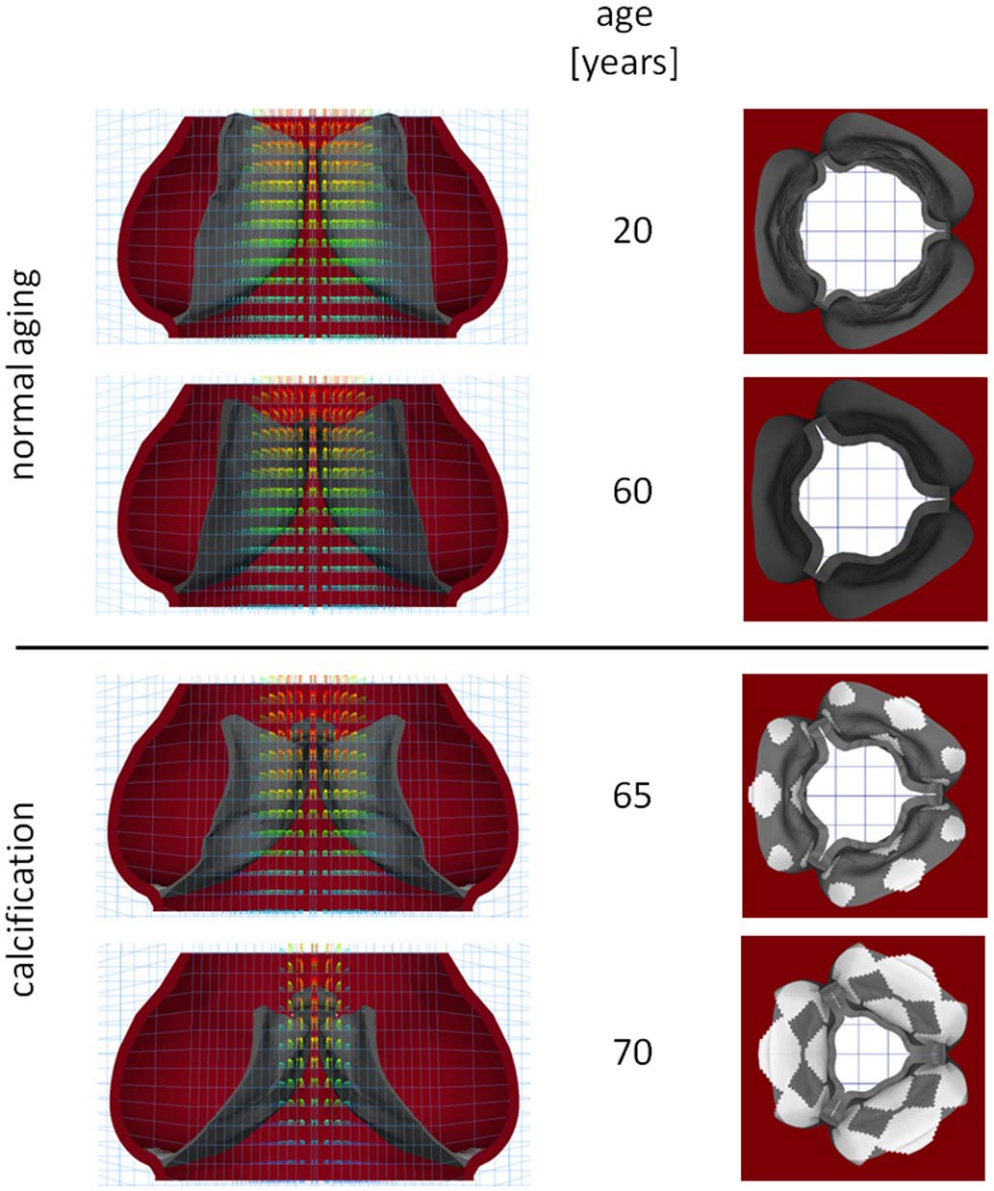
Computed geometries and flow velocities at mid-systole at various ages. Assumptions for calcification model are onset at age 50 and a growth rate of 1 mm/year.

In both normal aging and calcification, overall valve function, measured by fluid peak velocity and valve orifice area, degraded over time. Results are shown graphically in Figure 5, where the computed geometries are shown at mid-diastole for a range of ages. At each age, a section view overlaid with fluid velocity vectors as well as a view of the whole valve seen from the aortic orifice are shown. These plots show qualitatively the valve orifice narrowing both in normal aging and CAS.

**Figure 6:**
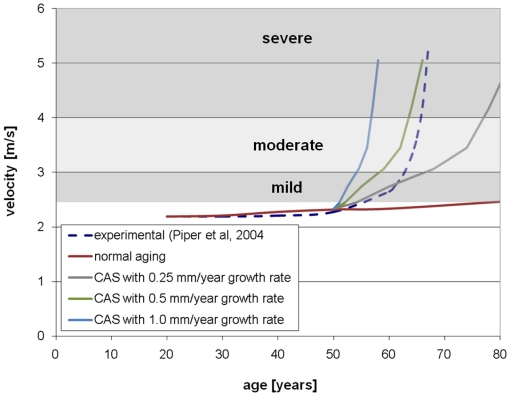
Peak velocity versus time for normal aging and with calcification.

**Figure 7:**
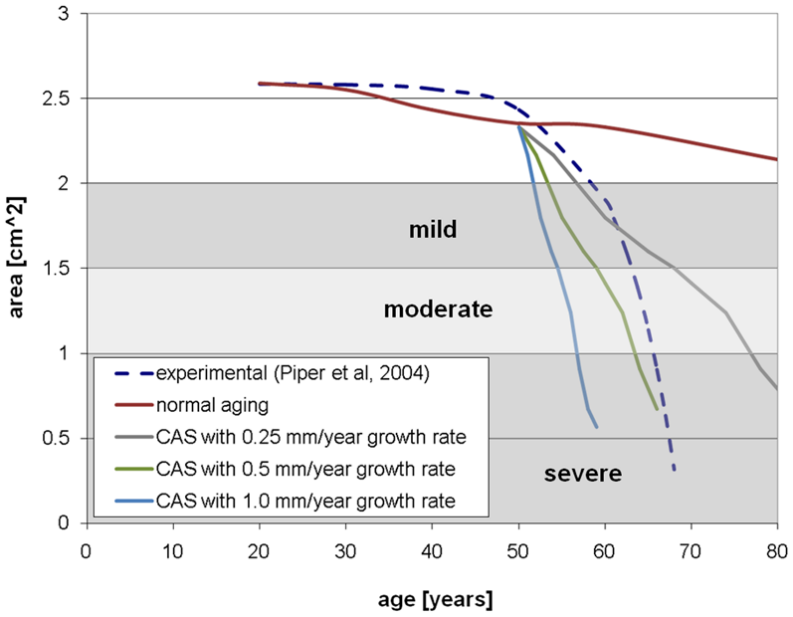
Valve effective area versus time for normal aging and with calcification.

Computed peak velocities and areas are compared to experimental data in Figure 6 and Figure 7, respectively. The theoretical curve in these figures has disease onset at 50 years and a range of growth rates. The experimental data is the typical curve for a patient in whom calcification appears at 50 years of age in a previously unobstructed valve [15]. The plots are overlaid with clinically accepted values for grading the severity of valve disease [19]. In both plots, the theoretical model tracks the experimental data best with lower growth rate in the years immediately after onset of calcification and with higher growth rate in the years after that.

**Figure 8:**
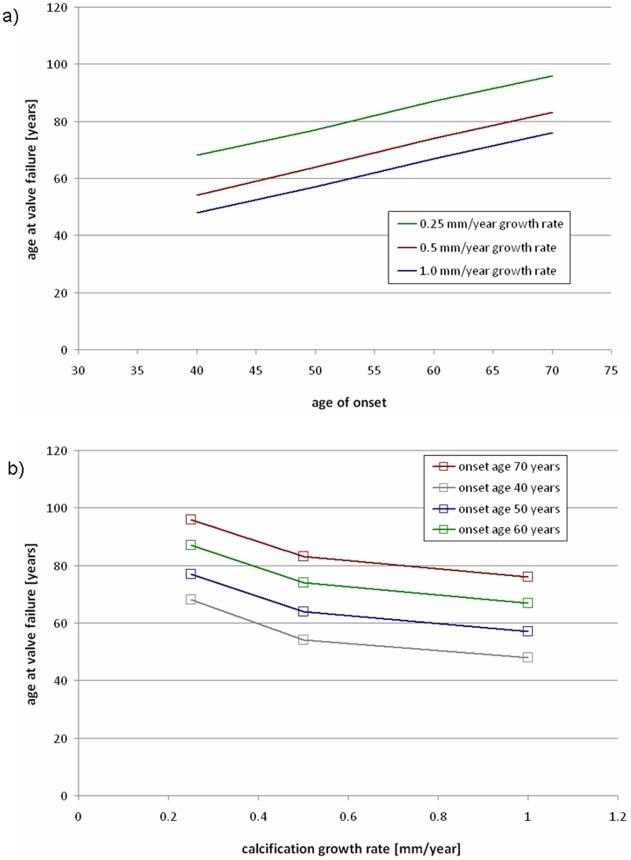
Sensitivity of model to input parameters. a) age where valve fails versus onset age, with various growth rates, and b) age where valve fails versus growth rate, with various ages of onset.

Sensitivity of the model to the two input parameters was analyzed. The age of valve failure, defined as the age when the AVA reached <1.0 cm^2^ [12], was recorded for all combinations of the input parameters. Figure 8.a shows the age where the valve fails versus the defined age of calcification onset, with curves for various growth rates. Figure 8.b shows the age at valve failure versus growth rate with various ages of onset, calculated at the same ages and rates as Figure 8.a.

## Discussion

We have developed a model for the mechanical consequences of aging in the AV, including normal stiffening and thickening as well as progressive calcification. This model predicts the organ-scale valve motion based on changes to the tissue-scale mechanical properties. As such, the simulation method described above has two directions for potential clinical translation. First, by incorporating the tissue-scale nature of calcification, the model may be able to more accurately predict the degradation of valve function than current methods. Second, the model may be used to evaluate treatments that aim to modify the tissue properties using input parameters consistent with a diminished rate of calcification owing to prevention or therapy.

There are limitations to this study and approximations made in this model. First, a number of assumptions are made to construct a model of the valve at any point in time. These assumptions, which we have previously discussed in detail [18], include simplified representation of the geometry, modeling the interaction of the valve with its environment through pressure and displacement boundary conditions, and assumptions inherent to the material models: a discrete fiber model for the leaflet mechanics, simple Mooney-Rivlin for the sinus wall, and Newtonian fluid for the blood.

Further assumptions were made to model the changes in the valve over time. In normal aging, the most significant source of error is our scaling of the leaflet extensibilities. Our model was extrapolated from the known data [2], which provides only the change in radial extensibility, and does not give reference to a no-stress state. Changes to the valve other than leaflet thickening and stiffening were not included in the model.

Calcification was modeled simply as the addition of stiff shell elements on the aortic surface. Calcification sites were assumed to appear simultaneously at different locations in the valve, where physiologically they arise at different times. The nodules were modeled as two-dimensional shells, though in CAS the calcifications are known to develop significant thickness.

Our current model assumes two input parameters, the age of calcification onset and the calcification growth rate. Sensitivity analysis shows that the model's ability to predict overall valve function, measured by when the model predicts valve failure will occur, is sensitive to both parameters. The current model is a simplification of the true three-dimensional, inhomogeneous progression of calcification observed in patients. We expect that future work will refine the growth model.

Our model provides a framework for linking overall valve function to valve mechanical properties and geometry. This model qualitatively captures the valve narrowing and increase in fluid velocity seen in patients. With proper choice of input parameters, the model can approximate experimental data for disease progression. If the age of onset and growth behavior, which likely does not follow the constant-rate model we have assumed, can be measured, then our model can be clinically useful. A model that can take patient inputs and predict the course of disease will be useful in deciding timing of valve replacement, and a model that can describe the effects of pharmaceutical and surgical interventions can aid in the development of those treatments. The model we have presented gives a theoretical basis for understanding the link between therapy and valve function in CAS and for being able to understand and predict the course of CAS can have significant clinical impact.

In order to construct an accurate description of the calcification growth, and thus to create a useful model of valve function, significant experimental work is required. Currently, the biomolecular processes involved in CAS and their link the changes in local mechanical properties are poorly understood. We can suggest an approach to creation of an accurate constitutive model of the spread of calcification. First, CAS is recognized to be a multiscale process, spanning scales from the molecular to the organ. Accordingly, a model of CAS pathology should span the relevant length scales. We have previously described the multiscale function of the healthy valve [18], [20], and this work should be extended to the diseased states. To construct such a model, multiscale experimental data on the disease process is required. While data on disease progression is difficult to collect in humans, existing small animal models for CAS, such as that in the rabbit [21], [22], can be utilized. In an animal model, mechanical and pathological changes can be observed across the length scales. For example, histology taken from animals sacrificed at varying ages with yield observation of the disease process at the cell and tissue scales, while live imaging can record organ-scale dynamic effects. An experimentally-based model of calcification progression at the smaller scales, coupled with the organ-scale methods described in this paper, will yield a useful predictive model for the disease CAS.

## Methods

We have previously described methods for using finite-element simulations to model the mechanical behavior of the normal aortic valve [18] and bicuspid aortic valve [20] over the cell, tissue, and organ length scales. These simulations have modeled the valve at one age in adulthood. In this paper, we extend our model of AV organ-scale mechanics to describe the transient effects of aging on the valve.

Here we briefly describe the method for modeling a healthy adult valve. Details on the development and validation of this model can be found in [18]. Valve geometry was created in SolidWorks (SolidWorks, Concord, MA). Dimensions of the overall valve structure [23] and thicknesses at various locations [24] were taken from literature. The geometry was meshed with brick elements using TrueGrid (XYZ Scientific Applications, Inc., Livermore, CA) and modified, including the addition of cable elements, using HyperMesh (Altair Engineering, Troy, MI). The cable elements are part of the discrete fiber model we developed [18] to model the highly nonlinear, anisotropic material behavior of the leaflets in a computationally efficient manner. Sinuses were modeled as isotropic and rubber-like, and blood was modeled as a simple Newtonian fluid. Dynamic pressure boundary conditions, representing the pressures in the left ventricle and aorta entrance, were applied to the blood at the valve orifices. A dynamic displacement boundary condition was applied to the ventricular orifice to represent ventricular contraction. The simulation was run in LS-DYNA (LSTC, Livermore, CA), which readily accepts large displacements of the solid through the fluid utilizing an operator-splitting algorithm [25]. Models were post-processed in HyperView (Altair Engineering, Troy, MI).

To simulate uncomplicated aging of the AV, individual simulations were created representing the valve at ages between 20 and 80 years at 10 year intervals. We are modeling the effects of aging on the adult valve, and have not included the ages from 0 to 20 where the young valve grows and remodels appreciably. Thicknesses were varied according to experimental data [1]. Thickening versus age data is shown in Figure 1.a and resulting CAD geometries of the valve at age 20 and 60 years are shown in Figure 1.b. Material properties were also varied following experimental data. The only known data for leaflet stiffening versus age is that of [2], plotted in Figure 2.a. We have previously discussed at length the choice of extensibilities in our model for the healthy AV [18]. Here, we consider that model to represent age 20. For all other ages, we scale the extensibilities proportionally following the linear fit illustrated in Figure 2.a. In Figure 2.b, we plot the resulting radial and circumferential stress-strain curves at ages 20 and 60.

Progression of CAS in the AV was modeled by adding calcified zones to the valve. Initiation sites for calcification were defined at the regions of high flexure (according to observations of explanted specimens), which occur along the attachment of the cusps to the aortic wall [26]. We have previously verified that our models predict highest flexure in the same regions as is generally observed [20]. These initial locations are shown in Figure 3. In the model, these calcified zones were modeled with stiff shell elements at the aortic-facing surface of the valve, the predominant site of clinical disease [27]. Our current disease progression model depends on two parameters. The first is the age of onset, at which calcified zones are first added to the model. Second, we assume a simple growth law for the calcific nodules: the boundary is allowed to spread outward at a constant speed. This speed, the growth rate, is the other parameter. We ran our model with onset ages from 40 to 70 years and growth rates from 0.25 mm/year to 1.0 mm/year. The growth rates were chosen to reflect the observed number of years required for calcification to progress fully across the cusp, from onset to valve failure [8], [15]. The percentage of the total leaflet area that is covered by calcification is plotted versus time in Figure 4 given a constant growth rate of 1 mm/year. With the boundary moving at a constant speed, the calcified area increases quadratically before saturating when the whole leaflet is covered. For each age of onset, the valve was simulated with each rate once for every year until the model valve was entirely calcified.

A number of measures of overall valve function and progression of valvular disease have been suggested, including peak fluid velocity, pressure drop across valve, effective orifice area, valve resistance [10], [11], [28], energy loss [29], rate of change in valve area [30], and others [13], [14]. To track the overall valve function over time in our simulations, we calculate the peak fluid velocity and aortic valve opening area (AVA) at each age. These measures were chosen because they are recognized clinically and straightforward to measure both in the clinic and simulation. Additionally, the area is an intrinsic measure of valve function, relatively insensitive to varying boundary conditions [28]. The peak velocity for each simulation is simply the maximum fluid velocity in the simulated cardiac cycle. AVA for each simulation is the maximum value of the area calculated throughout the cardiac cycle using the Gorlin formula [31]. Simulation results were compared to experimental data for a typical case of valve aging with CAS. Piper *et al*, 2004 gives experimentally-derived functions for AVA versus age given the calcification state of the valve at one point in time [15]. We compared our predicted AVA to the experimentally-determined curve for a valve which is unobstructed until onset of calcification at age 50 [15]. We also compared our predicted peak velocities to a curve calculated from the experimental AVA by the Gorlin formula.
